# Imaging Findings of Pediatric Rheumatic Disorders: JIA in the PRINTO Era and Autoimmune Interferonopathies

**DOI:** 10.1111/ped.70487

**Published:** 2026-08-03

**Authors:** Yuko Tsujioka, Yoshitake Yamada, Gen Nishimura, Tatsuo Kono, Masahiro Jinzaki

**Affiliations:** ^1^ Department of Radiology Keio University School of Medicine Tokyo Japan; ^2^ Department of Radiology Musashino‐Yowakai Hospital Tokyo Japan; ^3^ Department of Radiology Tokyo Metropolitan Children's Medical Center Tokyo Japan

**Keywords:** autoimmune interferonopathies, autoinflammatory–autoimmune continuum, juvenile idiopathic arthritis, pediatric musculoskeletal imaging, pediatric rheumatic diseases

## Abstract

Treatment strategies for pediatric rheumatic diseases have changed substantially over the past two decades, driven by the development of biologics and cytokine‐targeted molecular therapy. Therapeutic approaches that modulate both innate and adaptive immune responses have improved prognosis in these immune‐mediated disorders, and early diagnosis with timely intervention is associated with better outcomes. However, affected children often present with non‐specific symptoms, and reliable biomarkers remain limited in juvenile idiopathic arthritis (JIA); thus, the diagnostic delay remains common. Imaging is an important adjunct in pediatric rheumatology; in selected scenarios, it can be pivotal for establishing a definitive diagnosis and for assessing disease subtype, complications, and treatment response. This review presents an imaging‐focused overview of pediatric rheumatic diseases. We begin with a brief review of current immunologic concepts, including the autoinflammatory–autoimmune continuum, then illustrate typical imaging findings, discuss key differential diagnoses, and summarize the diagnostic strengths and limitations of major imaging modalities. We discuss in detail the major JIA subtypes based on the PRINTO classification, which aims to improve clinical coherence, facilitate risk stratification, and clarify relationships between pediatric and adult rheumatic entities. We also highlight interferonopathies—disorders characterized by a type I interferon signature—which provide a useful framework for understanding the mechanistic links between autoinflammation and autoimmunity along the continuum and for contextualizing selected autoimmune diseases, including systemic lupus erythematosus, juvenile dermatomyositis, and Sjögren's syndrome.

## 1 Introduction

Medical management of rheumatic diseases has been substantially transformed over the past two decades. In addition to conventional disease‐modifying antirheumatic drugs (DMARDs), particularly methotrexate (MTX), biologic therapies targeting innate immune cytokines have significantly improved clinical outcomes. Biologic therapy began with tumor necrosis factor‐α (TNF‐α) inhibition and subsequently expanded to other proinflammatory pathways, including interleukin (IL)‐1 and IL‐6, and more recently to the type I interferon (IFN‐I) axis. This evolution reflects increasing recognition that both aberrant innate immune activation and dysregulated adaptive immune responses contribute to disease onset and progression. More recently, therapeutic strategies have also expanded to cytokines that define helper T‐cell pathways (e.g., the IL‐17/IL‐23 axis).

These therapeutic advances have also been successfully implemented in pediatric rheumatology. However, diagnostic delay is more common in pediatric rheumatic diseases than in adulthood diseases because affected children often show non‐specific symptoms and an insidious onset, which increases the risk of physical morbidity. In addition, validated biomarkers for the most common juvenile idiopathic arthritis (JIA) remain limited. Therefore, imaging examination may play a primary role in the early diagnosis of affected children. In this review, we summarize imaging findings in pediatric rheumatic disorders, focusing on major JIA subtypes and autoimmune interferonopathies or autoimmune disorders associated with dysregulation of type I interferon. To facilitate understanding of the current advances in pathogenesis and therapy, we also briefly review monogenic rheumatic diseases due to dysregulated innate immunity and introduce the current immunologic framework for rheumatic diseases or “the concept of the autoinflammatory–autoimmune continuum.”

## 2 Immune Framework: Autoimmune‐Autoinflammatory Continuum

Many rheumatic diseases have traditionally been viewed through the lens of autoimmunity. Autoimmunity, conceptualized in the mid‐20th century, refers to dysregulated adaptive immunity in which loss of self‐tolerance drives autoreactive T‐ and B‐cell responses, often leading to autoantibody production and, in some diseases, immune‐complex–mediated tissue injury with secondary activation of innate immunity. Autoimmunity plays a major pathogenic role in common rheumatic diseases such as rheumatoid arthritis and systemic lupus erythematosus, which are therefore termed autoimmune diseases. In contrast, autoinflammation, recognized more prominently in the late 20th century, describes antigen‐independent, aberrant activation of innate immunity that induces cytokine‐driven inflammation with multi‐organ involvement and may secondarily engage adaptive immune responses, including autoimmunity. This framework emerged from studies of monogenic autoinflammatory diseases (e.g., familial Mediterranean fever and TNF receptor–associated periodic syndrome) and has since been extended to polygenic or acquired conditions such as gout, chronic noninfectious osteomyelitis (CNO)/chronic recurrent multifocal osteomyelitis (CRMO), and systemic juvenile idiopathic arthritis (JIA).

Both autoimmune and autoinflammatory diseases can involve a self‐perpetuating cycle of aberrant activation between the innate and adaptive immune systems, often conceptualized as an “autoinflammatory–autoimmune continuum.” This concept is further supported by the close link between autoimmunity and dysregulated type I interferon (IFN‐I) signaling. An “IFN signature,” reflecting upregulation of interferon‐stimulated genes, drives disease in monogenic interferonopathies (e.g., Aicardi–Goutières syndrome) and can amplify inflammation and autoimmunity in polygenic diseases such as systemic lupus erythematosus, juvenile dermatomyositis, and Sjögren syndrome.

## 3 Juvenile Idiopathic Arthritis: Epidemiology, Classification, and Imaging Overview

### 3.1 Epidemiology and Classification of JIA

Juvenile idiopathic arthritis (JIA) is the most common chronic rheumatic disease of childhood, with no identifiable cause. It is defined by disease onset before 16 years of age and arthritis persisting for at least six weeks. The incidence of JIA varies by region and ethnicity, ranging from 1.6 to 23 per 100,000 children [1]. Overall, JIA shows a female predominance; however, ESR‐JIA is more common in adolescent boys, and sJIA affects males and females approximately equally.

JIA is an umbrella term encompassing heterogeneous entities with distinct genetic backgrounds, immunopathology, and clinical phenotypes. The traditional International League of Associations for Rheumatology (ILAR) classification recognizes seven subtypes: systemic arthritis, oligoarthritis, RF‐negative polyarthritis, RF‐positive polyarthritis, psoriatic arthritis, enthesitis‐related arthritis, and undifferentiated arthritis [2]. However, this classification has been criticized because its phenotype‐based categorization does not adequately reflect the underlying pathophysiology and aligns poorly with adult rheumatic disease frameworks despite nosological similarities.

In contrast, the Pediatric Rheumatology International Trials Organization (PRINTO) proposed a pathobiology‐based framework comprising systemic JIA (sJIA), enthesitis/spondylitis‐related JIA (ESR‐JIA), RF‐positive JIA, early‐onset ANA‐positive JIA, and others (Table 1) [3]. This updated classification aims to improve clinical coherence and utility, facilitate risk stratification (e.g., increased uveitis risk in early‐onset ANA‐positive disease), and better align pediatric categories with adult counterparts. In this review, we discuss JIA in accordance with the PRINTO classification.

**TABLE 1:** PRINTO‐JIA classification (comparison with ILAR classification).

PRINTO category	PRINTO classification criteria	ILAR category corresponding to PRINTO	ILAR key definition	Adult counterpart
Systemic JIA	Fever and 2 major criteria or 1 major criterion and 2 minor criteria (major criteria: rash, arthritis, minor criteria: generalized lymphadenopathy, hepatosplenomegaly, serositis, arthralgia, leukocytosis)	Systemic arthritis	Arthritis + fever ≥ 2 weeks (quotidian ≥ 3 days) + ≥ 1 systemic feature (rash, lymphadenopathy, hepatosplenomegaly, serositis)	Adult‐onset Still's disease
RF‐positive JIA	Arthritis ≥ 6 weeks with RF positivity (typically ≥ 2 positive tests)	RF‐positive polyarthritis	Arthritis in ≥ 5 joints during the first 6 months + RF positive on ≥ 2 tests (≥ 3 months apart)	Seropositive rheumatoid arthritis (RA)
Enthesitis/spondylitis‐related JIA (ESR‐JIA)	arthritis and enthesitis, OR either of these plus any of the following (back pain, sacroiliitis on imaging, HLA‐B27 positivity, acute uveitis, SpA in a first‐degree relative)	Enthesitis‐related arthritis	Arthritis and enthesitis, or arthritis/enthesitis + ≥ 2 of SI tenderness/inflammatory back pain, HLA‑B27, onset in boy > 6, acute anterior uveitis, or family history of SpA/IBD‑related sacroiliitis	Spondyloarthritis (SpA)
Early‐onset ANA‐positive JIA	arthritis ≧ 6 weeks, age ≦ 6 years, and 2 positive ANA tests	Oligoarthritis and RF‐negative polyarthritis (subset)	ILAR splits these cases by joint count (oligo vs RF− poly); PRINTO re‐aggregates early‐onset ANA+ disease	No adult counterpart
Others	JIA phenotypes not meeting criteria for the above PRINTO categories (heterogeneous group)	Oligo/polyarthritis (RF−) and (ANA−); Psoriatic arthritis; Undifferentiated arthritis	Psoriatic: arthritis + psoriasis (or related features). Undifferentiated: overlap/does not fit categories	Seronagatice RA; Psoriatic arthritis

From an immunologic standpoint, these subtypes occupy distinct positions along an autoinflammatory–autoimmune continuum [4]. SJIA lies toward the autoinflammatory pole, whereas autoantibody‐positive JIA (RF‐positive JIA and early‐onset ANA‐positive JIA) lies toward the autoimmune pole. ESR‐JIA falls between these ends [5, 6]. Reflecting increasingly cytokine‐oriented insights into JIA pathogenesis, sJIA is considered an IL‐1/IL‐6 driven autoinflammatory disorder, whereas autoantibody‐positive JIA represents more “classical” adaptive immune–mediated disease. Consistent with their association with MHC class I alleles, ESR‐JIA and the spondyloarthritis‐like subset of psoriatic arthritis can be viewed as MHC‐I‐opathies, a group that also includes Behçet disease and enteropathic arthritis, and is linked to an IL‐23/IL‐17–centered pathway.

### 3.2 General View for Imaging Diagnosis for JIA

Imaging plays a pivotal role in confirming the presence of arthritis, defining disease subtypes, assessing complications, and monitoring the clinical course, including treatment response. It also aids the differential diagnosis of clinically similar conditions. The choice of imaging modality should be individualized to the patient's age, presenting features, and disease stage.

**FIGURE 1:**
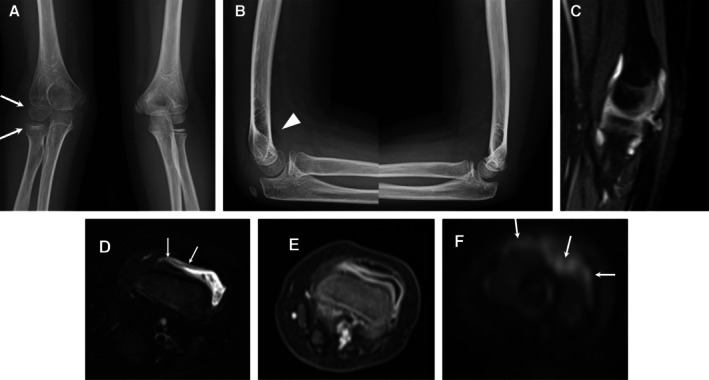
Two cases with ANA‐positive JIA. (A–C) A 7‐year‐old girl with mild pain and restricted extension of the right elbow joint that developed over the past 2 months. Frontal radiographs (A) revealed juxta‐articular osteopenia and epiphyseal overgrowth of the right elbow as compared with the left (arrows). Lateral radiographs (B) showed an elbow fat pad sign (arrowhead) indicating intra‐articular fluid collection. Fat‐suppressed T2‐weighted MR image (C) showed synovial thickening and joint effusion. (D–F) A 2‐year‐old girl with a 2‐month history of left knee pain, followed by joint contracture. Fat‐suppressed T2‐weighted image (D) showed high‐signal fluid accumulation and slightly lower‐signal synovial thickening (arrows). (E) Dynamic contrast‐enhanced MRI revealed early enhancement of the thickened synovium. (F) Diffusion‐weighted image showed restricted diffusion in the synovium (arrows).

**FIGURE 2:**
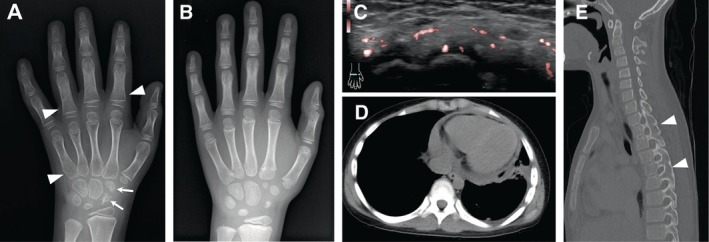
Two cases with late consequences of systemic JIA. (A–C) A 6‐year‐old boy with a long‐standing history of systemic JIA since 2 years of age. Frontal radiograph of the left hand (A) showed dysplastic carpal bones (arrows), mild metaphyseal widening of short tubular bones (arrowheads), and juxta‐articular osteopenia, compared with an age‐matched control (B). US (C) showed synovial thickening with increased power Doppler signal along tendon sheaths, suggesting active tenosynovitis/synovitis. (D, E) An 11‐year‐old boy with relapsed systemic JIA since 2 years of age. Non‐contrast‐enhanced CT showed pericardial and pleural effusions (D), and ankylosis of the posterior elements in the cervicothoracic spine (arrowheads) due to chronic inflammatory arthritis of the facet joints (E).

Plain radiography remains the initial study, which can exclude important mimics such as trauma and tumors, and may reveal indirect signs of joint abnormalities even at the early stage of JIA. These include soft tissue swelling, displacement of the periarticular fat pad (the “fat pad sign”, suggesting joint effusion), and cytokine‐mediated periarticular osteopenia (reflecting early inflammation) (Figure 1) [7]. When the insidious clinical course does not prompt medical attention over time, persistent hyperemia may lead to diffuse osteopenia, epiphyseal overgrowth, and leg‐length discrepancy due to overgrowth of the affected limb. Although transient overgrowth of affected limbs is typical in the early phase, prolonged inflammation can damage the physis, ultimately resulting in growth disturbance, often with final limb shortening. When interpreting subtle osseous changes, age‐dependent differences in ossification and bone modeling, as well as developmental variations, must be considered; comparison between the left and right sides is often useful. Given the relative thickness of pediatric articular cartilage, marginal bone erosion and joint space narrowing become apparent only in later stages. Joint ankylosis indicates a long‐standing disease (Figure 2).

Ultrasound (US) can demonstrate synovial hypertrophy, joint effusion, and tenosynovitis, and power Doppler US shows increased synovial or tendon‐sheath vascularity (Figure 2). US is well suited for early detection and short‐interval monitoring, as it can be performed at the bedside without sedation. The main limitation is a lesser capability to assess deep joints (e.g., the hip, sacroiliac joints, and temporomandibular joints), and the inability to evaluate bone marrow.

MRI provides the highest sensitivity for early synovial inflammation and enables a comprehensive assessment of ligaments, tendons, cartilage, and bone marrow. T2‐weighted imaging depicts synovial inflammation as synovial thickening with intermediate‐to‐high signal intensity, while contrast‐enhanced imaging demonstrates avid synovial enhancement, which can be further characterized with dynamic contrast‐enhanced sequences. Diffusion‐weighted imaging can further support the assessment of active synovitis (Figure 1) [8]. RI is particularly valuable for evaluating the atlantoaxial, Bone marrow edema adjacent to inflamed joints on fat‐suppressed T2‐weighted or STIR images can be seen in active arthritis and is considered a predictor of subsequent joint destruction [9]. MRI is particularly valuable for evaluating the atlantoaxial, subtalar, and temporomandibular joints and is essential for assessing axial disease, including the spine and sacroiliac joints. Drawbacks include the potential need for sedation and higher cost compared with US.

To assist pediatricians in selecting the most appropriate modality, the recommended imaging approach based on specific clinical findings, including the necessity of intravenous contrast, is summarized in Table 2. Generally, while contrast‐enhanced MRI remains the gold standard for assessing active synovitis, synovial thickening itself can also be detected on non‐contrast fluid‐sensitive sequences, although with lower sensitivity for inflammatory activity. In contrast, non‐contrast fluid‐sensitive sequences are usually sufficient for detecting bone marrow edema or myositis (Table 2).

**TABLE 2:** Practical imaging approach according to imaging targets in pediatric rheumatic diseases.

Clinical suspicion/target	Preferred modality	Contrast required?	Rationale/notes
Initial screening	Plain Radiography	No	Rule out fractures/tumors; assess growth abnormalities (overgrowth in the early stage and growth disturbance in the late stage)
Synovitis in superficial joints (e.g., fingers, wrists, knees, ankles)	Ultrasound	No	First‐line for superficial joints; power Doppler can evaluate vascularity (active inflammation) without sedation or contrast
Synovitis in deep joints (e.g., hips, shoulders, spine)	MRI	Yes (Recommended)	Contrast‐enhanced MRI remains the gold standard for assessing active synovitis; synovial thickening is detectable on T2‐weighted or STIR images, although non‐contrast MRI is less sensitive for active inflammation
Bone Marrow Edema	MRI (Fat‐suppressed T2WI/STIR)	No	Bone marrow edema on MRI is considered a predictor of subsequent structural joint damage
Enthesitis (Superficial)	Ultrasound (US)	No	Useful for assessing hypoechoic thickening and cortical irregularity
Sacroiliitis/Axial involvement	MRI	Usually no	Axial lesions develop insidiously years after disease onset and can progress to ankylosis.
Temporomandibular Joint Arthritis	MRI	Yes (Recommended)	TMJ involvement is often clinically silent and may progress insidiously, leading to mandibular growth disturbance and facial asymmetry; early synovitis and effusion are best detected with contrast‐enhanced MRI
Myositis	MRI	No	Fluid‐sensitive MRI sequences are highly sensitive for muscle edema

### 3.3 Region‐Specific Findings for the Cervical Spine and Temporomandibular Joint

**FIGURE 3:**
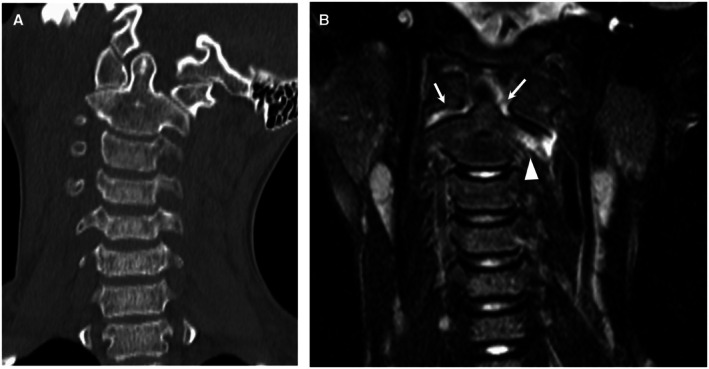
A 12‐year‐old girl with ANA‐positive JIA presenting with torticollis. (A) Coronal CT showed atlantoaxial rotatory subluxation. (B) Fat‐suppressed T2‐weighted coronal MRI showed atlantoaxial synovitis with increased signal (arrows) and bone marrow signal change in the left lateral mass of C2 (arrowhead).

Involvement of the cervical spine and temporomandibular joint (TMJ) may cause cervical myelopathy and difficulty with mastication; therefore, imaging of these regions deserves special comment. Children with RF‐positive JIA and long‐standing active disease are at risk for pannus formation around the odontoid process and atlantoaxial arthritis, which predisposes to atlantoaxial subluxation and may present clinically with torticollis and neurological signs. On plain radiography, the diagnosis of atlantoaxial instability rests on widening of the atlanto‐dental interval (ADI) or malalignment of C1/2. MRI can reveal mass‐like synovial thickening with contrast enhancement around the odontoid process, which may impinge on the spinal cord (Figure 3). MRI may also be used to evaluate the craniovertebral junction before general anesthesia to identify clinically occult abnormalities. TMJ synovitis is common, but often clinically silent, resulting in delayed diagnosis [10]. TMJ arthritis can impair mandibular condylar growth, leading to malocclusion and facial asymmetry. MRI enables early detection of synovitis, joint effusion, disc displacement, and condylar signal changes before skeletal deformity becomes apparent.

### 3.4 Clinical and Imaging Features by JIA Subtype

#### 3.4.1 Systemic JIA (sJIA)

Systemic JIA is considered the pediatric counterpart of adult‐onset Still's disease. Its pathogenesis is generally viewed as innate immune–dominant and skewed toward autoinflammation, with prominent cytokine dysregulation (e.g., IL‐1, IL‐6, and IL‐18) [11]. Historically, sJIA has been regarded as an autoinflammation‐dominant disease; however, genetic studies have identified strong associations with the MHC class II locus, including HLA‐DRB1*11, suggesting contributions beyond innate immunity [12].

Typical manifestations of sJIA include quotidian fever, an evanescent salmon‐pink rash, lymphadenopathy, hepatosplenomegaly, and serositis (often pericardial and/or pleural effusion) (Figure 2). Macrophage activation syndrome (MAS) is a potentially fatal complication characterized by hyperactivation of T‐cells and macrophages. Imaging findings are non‐specific and may include hepatosplenomegaly, gallbladder wall edema or thickening, ascites, and generalized lymphadenopathy.

A recently recognized entity, sJIA‐associated lung disease (sJIA‐LD), shows heterogeneous pathology often including mixed features of pulmonary alveolar proteinosis (PAP) and endogenous lipoid pneumonia (ELP), accompanied by lymphoplasmacytic inflammation. Reported risk factors include younger age at sJIA onset, prior or recurrent MAS, elevated IL‐18 levels, trisomy 21, and adverse reactions to biologic therapy [13, 14, 15]. High‐resolution CT typically demonstrates diffuse or patchy ground‐glass opacities with subpleural reticulation and interlobular septal thickening, sometimes presenting with a crazy‐paving pattern, which may be associated with mediastinal and/or hilar lymphadenopathy. Overall, this imaging pattern is consistent with the PAP/ELP spectrum. Chest CT is not recommended for routine screening and should be reserved for children with persistent or unexplained respiratory symptoms, abnormal initial pulmonary evaluation (chest radiography and/or pulmonary function testing), or other red‐flag features (e.g., new digital clubbing or drug hypersensitivity‐like reactions) [16].

Though classified under JIA, arthritis may not be the initial manifestation of sJIA, which distinguishes it from other JIA subtypes. Nevertheless, chronic arthritis becomes apparent over time. Arthritis in sJIA is characterized primarily by non‐erosive synovitis. Early imaging findings are largely non‐specific and resemble those of other types of JIA, including joint effusion, non‐erosive synovitis, and juxta‐articular soft tissue swelling and osteopenia. Over time, however, chronic systemic inflammation may lead to more characteristic skeletal sequelae, such as generalized osteoporosis and abnormal bone modeling (Figure 2).

Therapeutic management of sJIA remains challenging. Conventional DMARDs (e.g., MTX) have limited efficacy for systemic manifestations. Instead, IL‐1 and IL‐6 inhibitors are used as the first‐line of biologic therapies. Although many patients respond, control of arthritis may remain suboptimal, and approximately 40% of patients continue to have active arthritis and relapsing or refractory disease [17].

#### 3.4.2 RF‐Positive JIA

RF‐positive JIA represents the childhood‐onset counterpart of adult rheumatoid arthritis (RA) and typically affects adolescent girls. It is associated with the same HLA‐DRB1 alleles as adult RA [18]. Diagnostic criteria include arthritis persisting for at least six weeks and seropositivity for RF and/or anti–cyclic citrullinated peptide (anti‐CCP) antibodies, reflecting the predominant pathogenic role of adaptive immunity.

**FIGURE 4:**
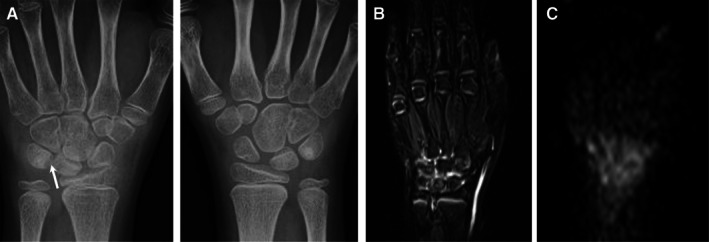
A 7‐year‐old girl with RF‐positive JIA. (A) Radiograph of both hands showed diffuse osteopenia, intercarpal joint space narrowing, and subchondral bone erosion of the lunate‐triquetrum joint of the left side (arrow), which is apparent compared with the less affected right side. (B) Fat‐suppressed T2‐weighted MRI showed intercarpal synovial thickening and increased signal intensities in the carpal bones. (C) Diffusion‐weighted image showed high signal intensity in the intercarpal joints.

The classic pattern of arthritis is bilateral symmetric polyarthritis involving the small joints of the hands and feet. Imaging often demonstrates marginal erosions and joint‐space narrowing of the interphalangeal joints and metacarpal joints with irregularity of the distal radioulnar joint and malalignment of the carpal rows (Figure 4). RF‐positive JIA is also frequently associated with atlantoaxial joint involvement and temporomandibular joint destruction. Extra‐articular manifestations such as rheumatoid nodules, vasculitic skin lesions, and rarely, interstitial lung disease may occur, particularly in chronic uncontrolled disease.

Early initiation of MTX and biologic approach targeting the TNF‐α or IL‐6 pathways is crucial for improved outcomes; however, despite aggressive therapy, remission is less frequent than in other JIA subtypes, and treatment can only rarely be discontinued within 5 years [19]. Recurrence is common even after remission, and radiographic damage persists in up to 45% of cases.

#### 3.4.3 Enthesitis/Spondylitis‐Related JIA

Enthesitis/spondylitis‐related JIA (ESR‐JIA) corresponds to the enthesitis‐related arthritis (ERA) category of the former ILAR classification and shows substantial genetic and clinical overlap with adult spondyloarthritis (SpA). In the PRINTO system, ESR‐JIA is categorized within the MHC‐I‐opathies, a group in which disease onset is thought to be driven primarily by innate immune mechanisms and MHC class I–related pathways. Conceptually, MHC‐I‐opathies occupy an intermediate position between autoimmunity and autoinflammation. Arthritis in MHC‐I‐opathies is related to the IL‐23/IL–17–centered pathway that is promoted by T cells resident at mechanically stressed entheses.

ESR‐JIA is characterized by arthritis with enthesitis, or defined by at least two of the following: sacroiliac (SI) joint tenderness, inflammatory back pain, HLA‐B27 positivity, acute anterior uveitis, or a family history of SpA. Because sacroiliitis may occur in IBD‐associated juvenile SpA (enteropathic arthritis), underlying Crohn disease or ulcerative colitis should be considered in children presenting with sacroiliitis. ESR‐JIA typically affects boys aged ≥ 6 years. Hallmark features include predominantly lower‐limb arthritis and truncal pain related to SI and axial skeleton involvement. Axial lesions may develop insidiously over several years after disease onset and are often preceded by appendicular joint involvement.

MRI is the preferred screening modality and offers the highest sensitivity for detecting SI joint involvement [7, 20]. Active SI lesions include subchondral bone marrow edema, synovitis, and capsulitis, whereas structural changes include erosions, fat metaplasia, and sclerosis [21, 22]. In the spine, MRI demonstrates Romanus‐type corner lesions and enthesitis at the facet joints or spinous processes. US can be used to assess inflammation of superficial entheses, which show hypoechoic thickening, cortical irregularity, and increased power Doppler signal. Late sequelae of the axial skeleton (e.g., bamboo spine and ankylosis of the SI joint) can be depicted on plain radiographs.

#### 3.4.4 Early‐Onset ANA‐Positive JIA

Early‐onset ANA‐positive JIA is a newly defined, distinct subtype in the PRINTO classification that enables more consistent categorization. Notably, it has no adult counterpart and represents a pediatric‐specific entity, characterized by oligoarthritis or polyarthritis with antinuclear antibody (ANA) positivity in children ≤ 6 years of age [23]. This subtype is characterized by autoantibody‐associated adaptive immune dysregulation and shows a strong association with HLA class II genes [24]. Its immunological background is distinct from that of MHC‐I‐opathies (e.g., ERA‐JIA).

The clinical hallmark of this subtype is asymmetric arthritis predominantly affecting large joints. Affected children present with joint swelling or morning stiffness but often with only mild pain. Chronic anterior uveitis is a major extra‐articular complication in ANA‐positive JIA. Often being asymptomatic but potentially vision‐threatening, this ocular involvement necessitates regular ophthalmologic screening for early detection and management [25].

Joint involvement is typically non‐erosive. In the early stage, plain radiographs show soft‐tissue swelling around the affected joint or joint distension and juxta‐articular osteopenia, whereas US and MRI typically show joint effusion and mild synovial hypertrophy. Structural damage is uncommon at this stage. With long‐standing uncontrolled disease, growth plate disturbance and limb‐length discrepancy develop, and in rare cases, marked epiphyseal overgrowth (“mega epiphysis”) may be seen (Figure 1). Even in the late stages, severe erosive change and ankylosis are less frequent than in RF‐positive JIA. MTX remains the cornerstone of treatment. In patients with high disease activity, a prolonged course, or associated uveitis, early initiation of a biologic approach may be beneficial.

## 4 Differential Diagnosis of JIA

The differential diagnosis of JIA, particularly early‐onset ANA‐positive JIA, encompasses a wide range of conditions that present with monoarthritis or oligoarthritis, such as infection, hematologic disorders, and tumor‐like lesions. In general, the differentiation is not so challenging based on clinical manifestations, the properties of aspirated joint fluid, and laboratory data.

Septic arthritis secondary to adjacent osteomyelitis may be clinically indistinguishable from the initial presentation of monoarticular JIA. Reflecting its acute onset, septic arthritis shows only non‐specific soft tissue swelling on plain radiographs. However, US can demonstrate marked capsular distension and may reveal a subperiosteal fluid collection suggestive of osteomyelitis. MRI can exhibit an intraosseous abscess, typically appearing as a small focus with marked hyperintensity on diffusion‐weighted imaging, accompanied by extensive inflammatory signal changes in the surrounding soft tissues.

Chronic nonbacterial osteitis/chronic recurrent multifocal osteomyelitis (CNO/CRMO) can clinically mimic JIA, but the differential diagnosis between both conditions is often clear on imaging grounds. The classical manifestation of CNO/CRMO is insidiously progressive metaphyseal osteitis, which appears as patchy lucent areas with surrounding sclerosis and periosteal reaction and is associated with bone marrow edema depicted on MRI. Over time, cortical thickening and subperiosteal new bone formation may develop in the meta‐diaphyseal regions [7, 26].

**FIGURE 5:**
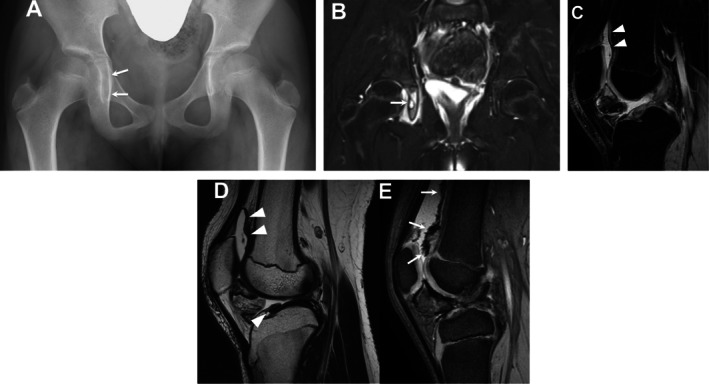
Differential diagnoses of JIA. (A, B) Osteoid osteoma of the right ischium in a 13‐year‐old girl with right hip pain. Radiograph of the hip (A) showed mild juxta‐articular osteopenia and enlargement of the acetabular teardrop (arrows). Fat‐suppressed T2‐weighted image (B) showed joint effusion, intraosseous and juxtaosseous edema of the acetabular teardrop (inferomedial aspect of the acetabulum), and a nidus of osteoid osteoma, seen as a well‐defined, high intensity focus surrounded by a low intensity rim (arrow). (C, D) Hemophilic arthropathy in a 13‐year‐old boy with painful knee swelling. Hemosiderin deposition along the synovial lining (arrowheads) was more clearly depicted on gradient‐echo T2‐weighted image (C) than on the spin echo T2‐weighted image (D). (E) Tenosynovial giant cell tumor in a 9‐year‐old boy with knee swelling. Hemosiderin deposition was depicted as low signal intensity with “blooming” on T2* gradient‐echo sequence (arrows).

Hemophilic arthropathy is characterized by intra‐articular hemosiderin deposition due to recurrent hemarthrosis, which can be delineated as markedly low signals within the synovial membrane on T2*‐weighted imaging [7]. Intra‐articular osteoid osteoma is associated with synovitis that mimics JIA. Cortical osteoid osteoma is characterized by a radiolucent nidus embedded in a florid periosteal reaction. In contrast, intra‐articular osteoid osteoma often demonstrates a small focus of radiolucent nidus, juxtaarticular osteopenia due to persistent synovial hyperemia, and subtle osteosclerosis. This constellation may be difficult to identify on plain radiographs but is readily discernible on CT and, in many cases, on MRI (Figure 5).

Children with leukemia commonly present with painful joints and initially show a normal cell count. Distinctive metaphyseal translucencies, so‐called leukemic lines, are an important diagnostic clue. MRI can delineate diffuse bone marrow infiltration, appearing as uniformly low signal intensities on T1‐weighted imaging. Malignant tumors, such as Ewing sarcoma and osteosarcoma, may present with symptoms that overlap with JIA; however, the differential diagnosis is usually straightforward on imaging grounds.

## 5 Interferonopathy: Exemplars Along the Continuum

Recently, aberrant activation of IFN‐I signaling, often referred to as an “IFN signature”, has attracted considerable attention in the pathogenesis of, and therapeutic approaches to, several rheumatic disorders. Similar to monogenic autoinflammatory diseases, genetic disorders with IFN signature have provided key insights into the relationship between autoinflammation and autoimmunity and serve as paradigmatic examples along the autoinflammatory–autoimmune continuum. IFN‐I (IFN‐α and IFN‐β) are central cytokines of the innate antiviral defense mechanism. A group of monogenic disorders is caused by aberrant upregulation of IFN‐I production in the absence of viral infection, which not only induces autoinflammation but also irrationally activates adaptive immunity and promotes autoimmunity. These disorders are collectively referred to as autoinflammatory interferonopathies.

A classic example of autoinflammatory interferonopathies is Aicardi–Goutières syndrome (AGS), a congenital leukoencephalopathy with brain calcification. Recognition of the clinical similarities between AGS and congenital TORCH prompted investigation into the pathogenic importance of IFN‐I in AGS and other interferonopathies, including Singleton‐Merten syndrome, STING‐associated vasculopathy with onset in infancy (SAVI), spondyloenchondrodysplasia, and Nakajo‐Nishimura syndrome (also termed chronic atypical neutrophilic dermatosis with lipodystrophy and elevated temperature (CANDLE)) [27]. Target organs in autoinflammatory interferonopathies include the brain, skin, vasculature, lungs, joints, and soft tissue, with corresponding inflammatory manifestations such as encephalopathy, chilblain‐like skin lesions, peripheral and intracerebral vasculopathy, interstitial lung disease, non‐erosive arthritis, and myopathy/lipodystrophy, respectively.

Most recently, the framework of interferonopathy has been extended into a subset of autoimmune disorders, such as SLE, dermatomyositis, and Sjögren syndrome. These disorders are commonly associated with IFN signature that likely exacerbates their clinical disease activity. In this context, dysregulated IFN‐I signaling is considered a driver of autoimmunity and an amplifier of inflammation rather than merely a secondary phenomenon. Such conditions have been referred to as autoimmune interferonopathies.

Janus kinase (JAK) inhibitors, which can attenuate IFN‐I signaling, have been widely used for both monogenic autoinflammatory interferonopathies and polygenic autoimmune interferonopathies. Although evidence remains incomplete, accumulating clinical experience supports their efficacy in rheumatologic practice.

In this review, we focus on the clinical and imaging findings of autoimmune interferonopathies. Autoinflammatory interferonopathies are rare and are not routinely encountered in general rheumatology practice; therefore, our discussion of these conditions is brief. Nonetheless, recognizing the phenotypic overlap between autoinflammatory and autoimmune interferonopathies may facilitate understanding of the clinical heterogeneity of rheumatic diseases.

## 6 Systemic Lupus Erythematosus (SLE)

SLE is a multisystem autoimmune disease in which IFN signature plays a major pathogenic role in disease progression. In some cohorts, approximately 7%–8% of children with SLE have a monogenic etiology, including mutations in genes implicated in autoinflammatory interferonopathies [28]. For example, mutations in *TREX1* cause a subtype of AGS (AGS type 1) and have also been identified in monogenic SLE. Childhood‐onset SLE tends to have a more aggressive clinical course and greater organ involvement than adult‐onset SLE. Major target organs include the skin, kidneys, serosal surfaces, joints, and central nervous system.

Given the multisystem involvement and the frequent occurrence of infection under immunosuppressive therapy, a multimodality approach is essential for imaging evaluation in affected children. Imaging is crucial for detecting organ‐specific abnormalities, grading the disease severity, excluding life‐threatening complications, and monitoring therapeutic response and adverse effects.

**FIGURE 6:**
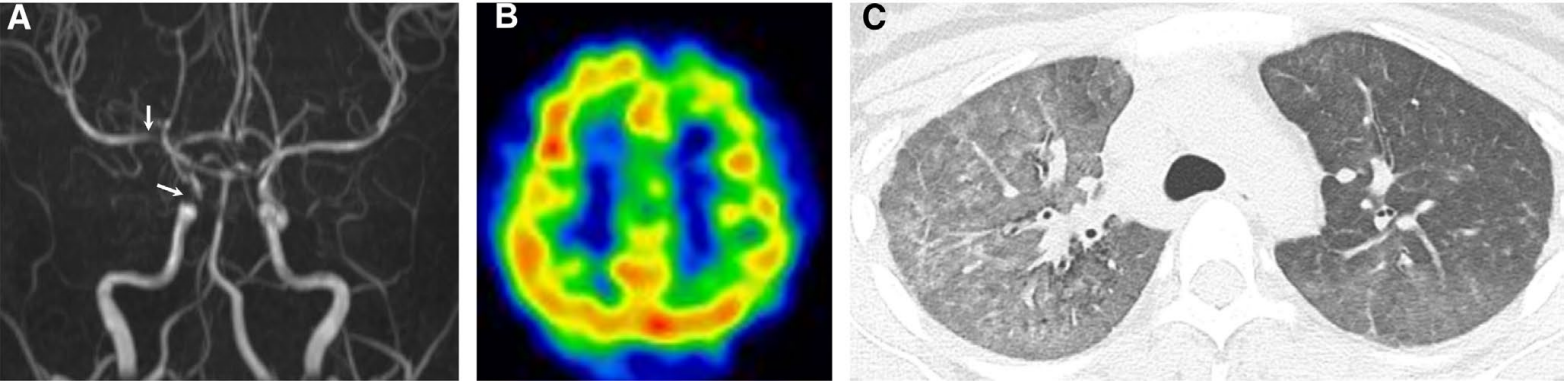
Two cases with SLE. (A, B) A 12‐year‐old girl with neuropsychiatric SLE presenting with severe headache. Conventional MRI showed no signal abnormalities on T1WI or T2WI (not shown). MR angiography (A) showed multifocal stenosis of the major intracranial arteries (arrows). 123I‐IMP brain perfusion SPECT (B) demonstrated multifocal decreased uptake in both cerebral hemispheres. (C) A 16‐year‐old girl with SLE complicated by diffuse alveolar hemorrhage. CT showed extensive, non‐segmental ground‐glass opacities with interlobular septal thickening in both lungs, more pronounced on the right.

MRI is essential to assess neuropsychiatric SLE (NPSLE), which may present with ischemic infarcts and hemorrhage. Cerebral venous sinus thrombosis is an important complication, particularly in patients with antiphospholipid syndrome. Additional imaging patterns include periventricular or juxtacortical white‐matter disease, posterior reversible encephalopathy syndrome, microbleeds detectable only on susceptibility‐weighted imaging, and, rarely, moyamoya‐like arterial stenoses (Figure 6A,B). Nonetheless, MRI is normal in a substantial proportion of patients with clinically active NPSLE, particularly those with non‐focal neurologic manifestations (e.g., headache, cognitive dysfunction, and mood disorders) [29].

Chest CT is important for evaluating pulmonary complications of SLE, particularly diffuse alveolar hemorrhage (DAH) and antiphospholipid syndrome–related pulmonary embolism. DAH typically appears as diffuse or patchy ground‐glass opacities with or without consolidation; superimposed septal thickening may produce a crazy‐paving pattern, and the distribution can change rapidly on short‐interval follow‐up (Figure 6C). CT angiography is used to confirm pulmonary embolism.

Although uncommon, interstitial lung disease (ILD) in SLE most often shows an NSIP pattern, characterized by bilateral ground‐glass opacities with fine reticulation, usually with lower‐lobe and subpleural predominance (sometimes with traction bronchiectasis). Acute lupus pneumonitis is rarer and typically shows multifocal or diffuse ground‐glass opacities and consolidation, often overlapping with infection.

Musculoskeletal involvement is typically characterized by non‐erosive synovitis. US reveals mild synovial hypertrophy with increased power Doppler signal, whereas MRI demonstrates synovial thickening with contrast enhancement and mild joint effusion. Osteonecrosis of the capital femoral epiphyses can occur in patients receiving long‐term glucocorticoid therapy and is best detected with MRI.

Lupus nephritis is a potentially life‐threatening complication that is primarily diagnosed on clinical grounds, and renal biopsy may be required for classification and prognostication. Advanced MRI techniques, such as diffusion tensor imaging (DTI), may provide adjunctive information in chronic kidney disease, including markers related to disease activity [30].

## 7 Juvenile Dermatomyositis (JDM)

JDM is the most common pediatric inflammatory myopathy, with most cases presenting between 5 and 14 years of age. Its key pathologic feature is an immune‐mediated small‐vessel vasculopathy that primarily involves the skin and skeletal muscle and, in a subset of patients, affects the lungs; this phenotype is thought to be closely linked to IFN signature.

**FIGURE 7:**
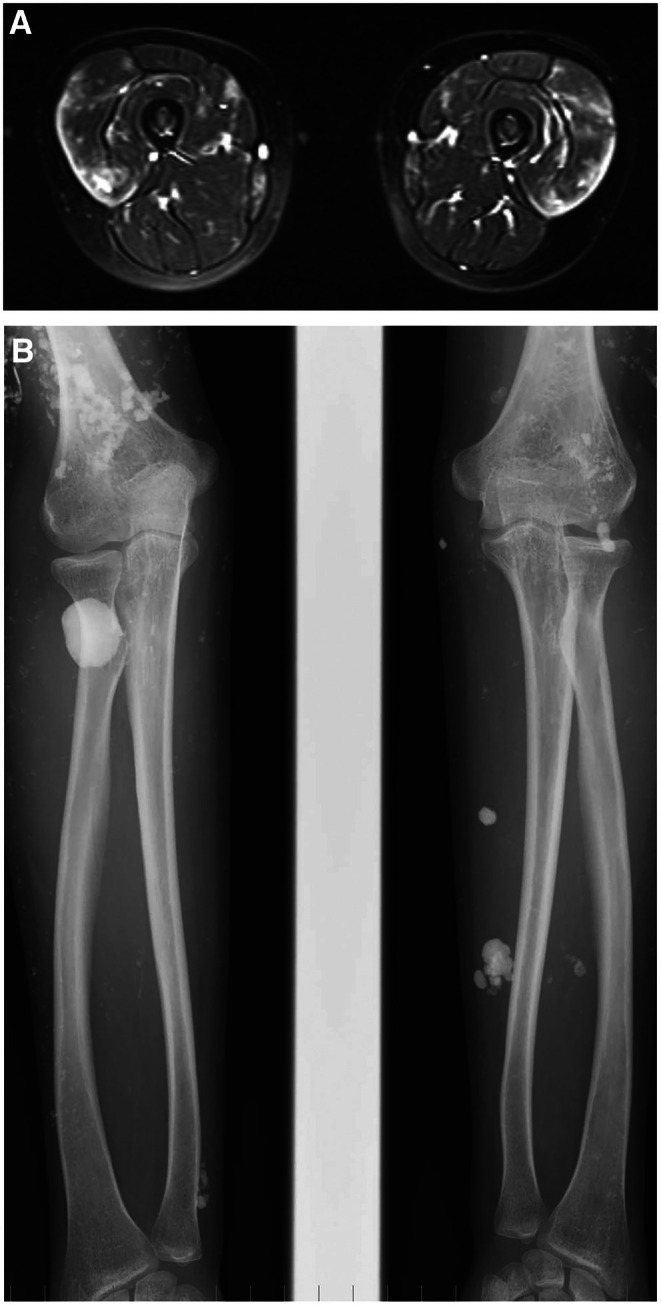
(A) A 6‐year‐old girl with juvenile dermatomyositis presenting with erythema and Gottron's papules without overt clinical symptoms of myositis. Fat‐suppressed T2‐weighted MRI showed bilateral hyperintensities in the thigh extensor muscles, suggestive of active myositis. (B) An adult male with multiple calcinosis lesions, who developed juvenile dermatomyositis at age 6. Multiple calcinosis lesions are present in the subcutaneous tissues of both forearms. The images were kindly provided by Dr. Taiki Nozaki, National Defense Medical College.

Unlike adults with DM, children with JDM are not at increased risk for malignancy. In contrast, JDM is more prone to subcutaneous calcinosis and acquired lipodystrophy, which can cause pain and physical morbidities [31]. MRI provides a noninvasive assessment of the activity of myositis. Active myositis is depicted as swollen muscle bellies with intramuscular T2 hyperintensities (Figure 7) (typically on fluid‐sensitive sequences), which evolved into muscular atrophy with intramuscular fatty replacement at the quiescent stage. Fascial and subcutaneous signal alterations may predict subsequent development of lipodystrophy or calcinosis [32, 33].

Pulmonary disease is generally less frequent in JDM than in adult DM. Pulmonary involvement ranges from insidiously progressive ILD to a potentially fatal entity termed rapidly progressive ILD (RP‐ILD). Children with JDM, particularly those with anti‐MDA5 antibodies, require vigilance for RP‐ILD even when respiratory symptoms are absent [34, 35]. RP‐ILD typically begins with peripheral/perilobular opacities in an organizing pneumonia (OP)‐like distribution, which can rapidly thicken and progress to diffuse ground‐glass opacities and consolidation, reflecting acute lung injury and diffuse alveolar damage. The background lung may be otherwise normal or may show NSIP/NSIP‐OP–like interstitial change.

HRCT‐based radiomics models have been used to predict the risks of ILD and RP‐ILD, as well as short‐term mortality of RP‐ILD in DM across both adult and pediatric cohorts. However, pediatric models specifically designed and validated for RP‐ILD remain scarce, representing an important avenue for further research [36, 37, 38].

## 8 Sjögren Syndrome (SS)

SS is an autoimmune “exocrinopathy” that typically manifests as dry mouth and dry eyes (Sicca symptoms). Given the consistent upregulation of interferon‐stimulated genes in blood and salivary glands and its association with immunologic activity, SS has increasingly been regarded as an autoimmune interferonopathy [39, 40]. Clinical symptoms and imaging findings differ between affected children and adults. In pediatric‐onset disease, sicca symptoms are often less prominent, whereas recurrent parotid swelling/parotitis is a common presenting feature. Systemic manifestations (e.g., fever, rash, arthralgia, lymphadenopathy) are relatively frequent, and pulmonary involvement may occur but appears less common in children than in adults [41].

US is the first choice of imaging evaluation for the salivary glands. In adults, US typically demonstrates parenchymal heterogeneity with multiple hypoechoic areas and, in more advanced disease, hyperechoic bands/foci related to chronic glandular damage. In affected children, parenchymal swelling and cystic changes are frequent, while hyperechoic bands and intraglandular fat are uncommon [42].

On MRI, particularly fat‐suppressed T2‐weighted imaging or MR sialography, multiple punctate hyperintense foci may be seen, reflecting punctate sialectasis (intraglandular ductal dilatation). The frequency and number of these foci tend to decrease with increasing age, while intraglandular fat is more characteristic of adult disease [42].

## 9 Novel Imaging Approaches for Pediatric Rheumatologic Diseases

Efforts to establish evidence‐based imaging approaches (e.g., standardized pediatric MRI protocols) have led to several published initiatives. An example is a JIA‐specific MRI scoring system that has been proposed to harmonize image acquisition and reporting across centers [43].

Although these techniques have been validated primarily in adult populations, advanced imaging techniques are conceptually appealing in pediatric rheumatic diseases. Quantitative cartilage imaging, particularly 3D T1ρ (T1rho) and T2 mapping, has shown potential to detect subtle cartilage and meniscal changes and can be utilized for longitudinal monitoring in both adults and children [44, 45].

Osseous changes of the axial skeleton are often assessed with CT. Novel cortical bone MRI techniques, such as ultrashort echo time (UTE) and zero echo time (ZTE) imaging, may reduce or eliminate the need for CT in selected cases and may be sufficient for evaluating subchondral bone changes around weight‐bearing joints [46]. Likewise, lung UTE MRI enables rapid, radiation‐free assessment of diffuse lung disease and may complement CT in long‐term follow‐up [47, 48, 49].

AI‐based optimization and quantitative analysis in adult rheumatic diseases have advanced rapidly across MRI applications, and similar progress is expected to improve workflow efficiency in pediatric joint MRI [50]. AI‐assisted dynamic contrast‐enhanced MRI techniques, such as an automatic selection of early post‐contrast phases and generation of voxel‐wise enhancement maps, can improve quantification of active synovitis in adult rheumatoid arthritis, and these methods may also apply to pediatric JIA cohorts, especially for objective monitoring within treat‐to‐target strategies [51, 52]. Similarly, deep learning–based reconstruction in MRI can substantially reduce radiation exposure while preserving image quality [53, 54, 55]. Similarly, deep learning–based reconstruction in MRI can shorten examination time while maintaining image quality, which is particularly advantageous in pediatric patients [56, 57].

## 10 Conclusion

This review provides an overview of the immunological background and imaging diagnosis of pediatric rheumatic diseases. Appropriate imaging assessment supports diagnosis and can reveal clinically silent lesions, help predict prognosis, and enable longitudinal monitoring of disease activity over time. Recent therapeutic advances have been guided, in part, by the concept of the autoinflammatory–autoimmune continuum, which has contributed to the development of multiple targeted therapies. In the current era of many treatment options, a treat‐to‐target strategy—adjusting therapy toward predefined clinical goals—has become increasingly important, and imaging should also be integrated into patient assessment and follow‐up in pediatric rheumatology.

## Author Contributions

Y.T. wrote this manuscript. Y.Y., G.N., T.K., and M.J. contributed to critical discussions and edited the manuscript. All authors have read and approved the final version of the manuscript and agree to its submission.

## Funding

The authors have nothing to report.

## Conflicts of Interest

The authors declare no conflicts of interest.

## Data Availability

Research data are not shared.

## References

[1] N. Bansal , C. Pasricha , P. Kumari , S. Jangra , R. Kaur , and R. Singh , “A Comprehensive Overview of Juvenile Idiopathic Arthritis: From Pathophysiology to Management,” Autoimmunity Reviews 22, no. 7 (2023): 103337. 37068698 10.1016/j.autrev.2023.103337

[2] R. E. Petty , T. R. Southwood , P. Manners , et al., “International League of Associations for Rheumatology Classification of Juvenile Idiopathic Arthritis: Second Revision, Edmonton, 2001,” Journal of Rheumatology 31, no. 2 (2004): 390–392. 14760812

[3] A. Martini , A. Ravelli , T. Avcin , et al., “Toward New Classification Criteria for Juvenile Idiopathic Arthritis: First Steps, Pediatric Rheumatology International Trials Organization International Consensus,” Journal of Rheumatology 46, no. 2 (2019): 190–197. 30275259 10.3899/jrheum.180168

[4] D. McGonagle , A. Aziz , L. J. Dickie , and M. F. McDermott , “An Integrated Classification of Pediatric Inflammatory Diseases, Based on the Concepts of Autoinflammation and the Immunological Disease Continuum,” Pediatric Research 65 (2009): 38R–45R. 10.1203/PDR.0b013e31819dbd0a 19190531

[5] E. D. Mellins , C. Macaubas , and A. A. Grom , “Pathogenesis of Systemic Juvenile Idiopathic Arthritis: Some Answers, More Questions,” Nature Reviews Rheumatology 7, no. 7 (2011): 416–426. 21647204 10.1038/nrrheum.2011.68 PMC4180659

[6] J. J. Kuiper , J. C. Prinz , E. Stratikos , et al., “EULAR Study Group on ‘MHC‐I‐Opathy’: Identifying Disease‐Overarching Mechanisms Across Disciplines and Borders,” Annals of the Rheumatic Diseases 82, no. 7 (2023): 887–896. 36987655 10.1136/ard-2022-222852 PMC10313995

[7] Y. Tsujioka , G. Nishimura , H. Sugimoto , T. Nozaki , T. Kono , and M. Jinzaki , “Imaging Findings of Juvenile Idiopathic Arthritis and Autoinflammatory Diseases in Children,” Japanese Journal of Radiology 41, no. 11 (2023): 1186–1207. 37329408 10.1007/s11604-023-01447-6 PMC10613601

[8] A. M. Barendregt , E. C. van Gulik , C. Lavini , et al., “Diffusion‐Weighted Imaging for Assessment of Synovial Inflammation in Juvenile Idiopathic Arthritis: A Promising Imaging Biomarker as an Alternative to Gadolinium‐Based Contrast Agents,” European Radiology 27, no. 11 (2017): 4889–4899, 10.1007/s00330-017-4876-y. 28608162 PMC5635098

[9] M. Mazzoni , A. Pistorio , F. Magnaguagno , et al., “Predictive Value of Magnetic Resonance Imaging in Patients With Juvenile Idiopathic Arthritis in Clinical Remission,” Arthritis Care and Research 75 (2023): 198–205, 10.1002/acr.24757. 34286915 PMC10087925

[10] C. J. Kellenberger , T. Junhasavasdikul , M. Tolend , and A. S. Doria , “Temporomandibular Joint Atlas for Detection and Grading of Juvenile Idiopathic Arthritis Involvement by Magnetic Resonance Imaging,” Pediatric Radiology 48, no. 3 (2018): 411–426. 29134239 10.1007/s00247-017-4000-0 PMC5823950

[11] N. Toplak , Š. Blazina , and T. Avčin , “The Role of IL‐1 Inhibition in Systemic Juvenile Idiopathic Arthritis: Current Status and Future Perspectives,” Drug Design, Development and Therapy 8, no. 12 (2018): 1633–1643. 10.2147/DDDT.S114532 PMC5996857 29922038

[12] M. J. Ombrello , V. L. Arthur , E. F. Remmers , et al., “Genetic Architecture Distinguishes Systemic Juvenile Idiopathic Arthritis From Other Forms of Juvenile Idiopathic Arthritis: Clinical and Therapeutic Implications,” Annals of the Rheumatic Diseases 76, no. 5 (2017): 906–913. 27927641 10.1136/annrheumdis-2016-210324 PMC5530341

[13] G. S. Schulert , S. Yasin , B. Carey , et al., “Systemic Juvenile Idiopathic Arthritis‐Associated Lung Disease: Characterization and Risk Factors,” Arthritis & Rhematology 71, no. 11 (2019): 1943–1954. 10.1002/art.41073 PMC6817389 31379071

[14] V. E. Saper , G. Chen , G. H. Deutsch , et al., “Emergent High Fatality Lung Disease in Systemic Juvenile Arthritis,” Annals of the Rheumatic Diseases 78, no. 12 (2019): 1722–1731. 31562126 10.1136/annrheumdis-2019-216040 PMC7065839

[15] D. Petrongari , P. Di Filippo , F. Misticoni , et al., “Lung Involvement in Systemic Juvenile Idiopathic Arthritis: A Narrative Review,” Diagnostics (Basel) 12, no. 12 (2022): 3095. 36553101 10.3390/diagnostics12123095 PMC9777523

[16] H. Wobma , R. Bachrach , J. Farrell , et al., “Development of a Screening Algorithm for Lung Disease in Systemic Juvenile Idiopathic Arthritis,” ACR Open Rheumatology 5, no. 10 (2023): 556–562. 37688362 10.1002/acr2.11600 PMC10570670

[17] G. Janow , L. E. Schanberg , S. Setoguchi , et al., “The Systemic Juvenile Idiopathic Arthritis Cohort of the Childhood Arthritis and Rheumatology Research Alliance Registry: 2010–2013,” Journal of Rheumatology 43, no. 9 (2016): 1755–1762. 27307527 10.3899/jrheum.150997

[18] A. O. Hersh and S. Prahalad , “Immunogenetics of Juvenile Idiopathic Arthritis: A Comprehensive Review,” Journal of Autoimmunity 64 (2015): 113–124. 26305060 10.1016/j.jaut.2015.08.002 PMC4838197

[19] J. Guzman , K. Oen , L. B. Tucker , et al., “The Outcomes of Juvenile Idiopathic Arthritis in Children Managed With Contemporary Treatments: Results From the ReACCh‐Out Cohort,” Annals of the Rheumatic Diseases 74, no. 10 (2015): 1854–1860. 24842571 10.1136/annrheumdis-2014-205372

[20] P. F. Weiss , R. Xiao , D. M. Biko , and N. A. Chauvin , “Assessment of Sacroiliitis at Diagnosis of Juvenile Spondyloarthritis by Radiography, Magnetic Resonance Imaging, and Clinical Examination,” Arthritis Care & Research (Hoboken) 68, no. 2 (2016): 187–194. 10.1002/acr.22665 PMC4720597 26212574

[21] T. M. Otobo , P. G. Conaghan , W. P. Maksymowych , et al., “Preliminary Definitions for Sacroiliac Joint Pathologies in the OMERACT Juvenile Idiopathic Arthritis Magnetic Resonance Imaging Score (OMERACT JAMRIS‐SIJ),” Journal of Rheumatology 46, no. 9 (2019): 1192–1197. 30770500 10.3899/jrheum.181115

[22] T. Nozaki , Y. Tsujioka , H. Sugimoto , et al., “Pearls and Pitfalls in Imaging of Axial Spondyloarthritis for Rheumatologists,” Modern Rheumatology 35, no. 4 (2025): 612–625. 40315052 10.1093/mr/roaf034

[23] B. Küçükali , Ç. Yıldız , B. T. Gülle , D. Gezgin Yıldırım , and S. A. Bakkaloğlu , “Evaluation of ILAR and PRINTO Classifications for Juvenile Idiopathic Arthritis: Oligoarticular JIA vs Early‐Onset ANA‐Positive JIA,” Clinical Rheumatology 44, no. 3 (2025): 1307–1316, 10.1007/s10067-025-07340-z. 39883305 PMC11865100

[24] L. N. Zaripova , A. Midgley , S. E. Christmas , M. W. Beresford , E. M. Baildam , and R. A. Oldershaw , “Juvenile Idiopathic Arthritis: From Aetiopathogenesis to Therapeutic Approaches,” Pediatric Rheumatology Online Journal 19, no. 1 (2021): 135. 34425842 10.1186/s12969-021-00629-8 PMC8383464

[25] J. W. van Straalen , G. Giancane , Y. Amazrhar , et al., “A Clinical Prediction Model for Estimating the Risk of Developing Uveitis in Patients With Juvenile Idiopathic Arthritis,” Rheumatology (Oxford, England) 60, no. 6 (2021): 2896–2905. 33274366 10.1093/rheumatology/keaa733 PMC8213427

[26] H. Himuro , S. Kurata , S. Nagata , et al., “Imaging Features in Patients With SAPHO/CRMO: A Pictorial Review,” Japanese Journal of Radiology 38, no. 7 (2020): 622–629. 32356235 10.1007/s11604-020-00953-1

[27] Y. J. Crow and N. Manel , “Aicardi–Goutières Syndrome and the Type I Interferonopathies,” Nature Reviews. Immunology 15, no. 7 (2015): 429–440. 10.1038/nri3850 26052098

[28] I. T. W. Harley and A. H. Sawalha , “Systemic Lupus Erythematosus as a Genetic Disease,” Clinical Immunology 236 (2022): 108953. 35149194 10.1016/j.clim.2022.108953 PMC9167620

[29] D. J. Tunnicliffe , D. Singh‐Grewal , S. Kim , J. C. Craig , and A. Tong , “Diagnosis, Monitoring, and Treatment of Systemic Lupus Erythematosus: A Systematic Review of Clinical Practice Guidelines: SLE Treatment Guidelines,” Arthritis Care & Research 67, no. 10 (2015): 1440–1452. 25778500 10.1002/acr.22591

[30] A. A. K. A. Razek , A. M. A. Khalek , S. Tharwat , M. K. Nassar , and N. Tharwat , “Diffusion Tensor Imaging of Renal Cortex in Lupus Nephritis,” Japanese Journal of Radiology 39, no. 11 (2021): 1069–1076. 34125367 10.1007/s11604-021-01154-0

[31] S.‐J. Na , S. M. Kim , I. N. Sunwoo , and Y.‐C. Choi , “Clinical Characteristics and Outcomes of Juvenile and Adult Dermatomyositis,” Journal of Korean Medical Science 24, no. 4 (2009): 715–721. 19654958 10.3346/jkms.2009.24.4.715 PMC2719214

[32] N. Sakurai , A. Hino‐Shishikura , T. Nozawa , et al., “Clinical Significance of Subcutaneous Fat and Fascial Involvement in Juvenile Dermatomyositis,” Modern Rheumatology 29, no. 5 (2019): 808–813. 30092673 10.1080/14397595.2018.1511026

[33] A. Bingham , G. Mamyrova , K. I. Rother , et al., “Predictors of Acquired Lipodystrophy in Juvenile‐Onset Dermatomyositis and a Gradient of Severity,” Medicine (Baltimore) 87, no. 2 (2008): 70–86. 18344805 10.1097/MD.0b013e31816bc604 PMC2674585

[34] P. Vignesh , P. L. Nadig , S. Basu , et al., “Profile of Patients With Juvenile Dermatomyositis and Anti‐MDA5 Autoantibodies,” Pediatric Research 97, no. 6 (2025): 2020–2028. 39313554 10.1038/s41390-024-03551-3

[35] Y. Tsujioka , G. Nishimura , E. Nishi , et al., “Childhood Interstitial Lung Diseases: Current Understanding of the Classification and Imaging Findings,” Japanese Journal of Radiology 42, no. 9 (2024): 937–952. 39012450 10.1007/s11604-024-01603-6 PMC11364587

[36] A. Haga , T. Iwasawa , T. Misumi , et al., “Correlation of CT‐Based Radiomics Analysis With Pathological Cellular Infiltration in Fibrosing Interstitial Lung Diseases,” Japanese Journal of Radiology 42, no. 10 (2024): 1157–1167. 38888852 10.1007/s11604-024-01607-2 PMC11442537

[37] Y. Li , W. Deng , Y. Zhou , et al., “A Nomogram Based on Clinical Factors and CT Radiomics for Predicting Anti‐MDA5+ DM Complicated by RP‐ILD,” Rheumatology (Oxford, England) 63, no. 3 (2024): 809–816. 37267146 10.1093/rheumatology/kead263

[38] L. Liu , M. Hu , Y. Zhou , et al., “Application of HRCT‐Based Radiomics to Predict Interstitial Lung Disease for Juvenile Dermatomyositis,” BMC Pediatrics 25, no. 1 (2025): 589. 40753199 10.1186/s12887-025-05968-z PMC12317514

[39] I. L. A. Bodewes , S. Al‐Ali , C. G. van Helden‐Meeuwsen , et al., “Systemic Interferon Type I and Type II Signatures in Primary Sjögren's Syndrome Reveal Differences in Biological Disease Activity,” Rheumatology (Oxford, England) 57, no. 5 (2018): 921–930. 29474655 10.1093/rheumatology/kex490

[40] N. Del Papa , A. Minniti , M. Lorini , et al., “The Role of Interferons in the Pathogenesis of Sjögren's Syndrome and Future Therapeutic Perspectives,” Biomolecules 11, no. 2 (2021): 251. 33572487 10.3390/biom11020251 PMC7916411

[41] R. L. Randell and S. M. Lieberman , “Unique Aspects of Pediatric Sjögren Disease,” Rheumatic Diseases Clinics of North America 47, no. 4 (2021): 707–723. 34635300 10.1016/j.rdc.2021.07.008 PMC8817684

[42] Y. Takagi , M. Sasaki , S. Eida , et al., “Comparison of Salivary Gland MRI and Ultrasonography Findings Among Patients With Sjögren's Syndrome Over a Wide Age Range,” Rheumatology (Oxford, England) 61, no. 5 (2022): 1986–1996. 34398226 10.1093/rheumatology/keab560 PMC9071520

[43] M. Navallas , M. Tolend , T. M. Otobo , et al., “Developing Standards for MRI Evaluation of Joints in Children With Juvenile Idiopathic Arthritis Utilizing the Temporomandibular Joint as a Model,” Japanese Journal of Radiology 42, no. 1 (2024): 56–68. 37626169 10.1007/s11604-023-01479-y

[44] W. C. Bae , V. Malis , Y. Kassai , and M. Miyazaki , “3D T1rho Sequences With FASE, UTE, and MAPSS Acquisitions for Knee Evaluation,” Japanese Journal of Radiology 41, no. 11 (2023): 1308–1315. 37247122 10.1007/s11604-023-01453-8 PMC11578038

[45] A. M. Barendregt , V. Mazzoli , J. M. van den Berg , et al., “T1ρ‐Mapping for Assessing Knee Joint Cartilage in Children With Juvenile Idiopathic Arthritis—Feasibility and Repeatability,” Pediatric Radiology 50, no. 3 (2020): 371–379. 31707445 10.1007/s00247-019-04557-4 PMC7026305

[46] K. Tsuchiya , M. Gomyo , S. Katase , S. Hiraoka , and H. Tateishi , “Magnetic Resonance Bone Imaging: Applications to Vertebral Lesions,” Japanese Journal of Radiology 41, no. 11 (2023): 1173–1185. 37209299 10.1007/s11604-023-01449-4 PMC10613598

[47] J. Geiger , K. G. Zeimpekis , A. Jung , A. Moeller , and C. J. Kellenberger , “Clinical Application of Ultrashort Echo‐Time MRI for Lung Pathologies in Children,” Clinical Radiology 76, no. 9 (2021): 708. 10.1016/j.crad.2021.05.015 34120734

[48] D. M. Renz , K.‐H. Herrmann , M. Kraemer , et al., “Ultrashort Echo Time MRI of the Lung in Children and Adolescents: Comparison With Non‐Enhanced Computed Tomography and Standard Post‐Contrast T1w MRI Sequences,” European Radiology 32, no. 3 (2022): 1833–1842. 34668994 10.1007/s00330-021-08236-7 PMC8831263

[49] H. Hatabu , M. Yanagawa , Y. Yamada , et al., “Recent Trends in Scientific Research in Chest Radiology: What to Do or Not to Do? That Is the Critical Question in Research,” Japanese Journal of Radiology 43, no. 6 (2025): 883–902. 39815124 10.1007/s11604-025-01735-3 PMC12125149

[50] S. Fujita , Y. Fushimi , R. Ito , et al., “Advancing Clinical MRI Exams With Artificial Intelligence: Japan's Contributions and Future Prospects,” Japanese Journal of Radiology 43, no. 3 (2025): 355–364. 39548049 10.1007/s11604-024-01689-y PMC11868336

[51] Y. Mori and N. Mori , “Selection of the Phase of Dynamic Contrast‐Enhanced Magnetic Resonance Imaging and Use of the Voxel‐Based Enhancement Maps May Facilitate the Assessment of Clinical Disease Activity in Patients With Rheumatoid Arthritis,” Japanese Journal of Radiology 43, no. 1 (2025): 138–139. 38913285 10.1007/s11604-024-01620-5

[52] W. Fang , Y. Mao , H. Wang , et al., “Fully Automatic Quantification for Hand Synovitis in Rheumatoid Arthritis Using Pixel‐Classification‐Based Segmentation Network in DCE‐MRI,” Japanese Journal of Radiology 42, no. 10 (2024): 1187–1197. 38789911 10.1007/s11604-024-01592-6

[53] J. Sun , H. Li , J. Li , et al., “Improving the Image Quality of Pediatric Chest CT Angiography With Low Radiation Dose and Contrast Volume Using Deep Learning Image Reconstruction,” Quantitative Imaging in Medicine and Surgery 11, no. 7 (2021): 3051–3058. 34249634 10.21037/qims-20-1158 PMC8250028

[54] S. L. Brady , A. T. Trout , E. Somasundaram , C. G. Anton , Y. Li , and J. R. Dillman , “Improving Image Quality and Reducing Radiation Dose for Pediatric CT by Using Deep Learning Reconstruction,” Radiology 298, no. 1 (2021): 180–188. 33201790 10.1148/radiol.2020202317

[55] T. Kaga , Y. Noda , T. Mori , et al., “Unenhanced Abdominal Low‐Dose CT Reconstructed With Deep Learning‐Based Image Reconstruction: Image Quality and Anatomical Structure Depiction,” Japanese Journal of Radiology 40, no. 7 (2022): 703–711. 35286578 10.1007/s11604-022-01259-0 PMC9252942

[56] N. Nishioka , Y. Shimizu , Y. Kaneko , et al., “Accelerating FLAIR Imaging via Deep Learning Reconstruction: Potential for Evaluating White Matter Hyperintensities,” Japanese Journal of Radiology 43, no. 2 (2025): 200–209. 39316286 10.1007/s11604-024-01666-5 PMC11790734

[57] S.‐H. Kim , Y. H. Choi , J. S. Lee , et al., “Deep Learning Reconstruction in Pediatric Brain MRI: Comparison of Image Quality With Conventional T2‐Weighted MRI,” Neuroradiology 65, no. 1 (2023): 207–214. 36156109 10.1007/s00234-022-03053-1

